# Supplementary material for: MetaRibo-Seq measures translation in microbiomes
Source: Nat Commun. 2020 Jun 29;11:3268. doi: 10.1038/s41467-020-17081-z (PMC7324362; doi:10.1038/s41467-020-17081-z)
Supplement: Supplementary file 10 — Supplementary Data 7 [file 41467_2020_17081_MOESM10_ESM.zip › File2/Confidence_High_Taxonomy/142861_out.krona.html]

Javascript must be enabled to view this page.

members
magnitude
magnitudeUnassigned
count
unassigned
taxon
rank

142861\_out

4

4
2
superkingdom

phylum
201174
2

2
class
1760

2
order
85012

2
2004
family

2
28443
genus

688067
species
2

SRS063040\_contig\_number\_contig-100\_20599.153383SRS140513\_contig\_number\_967

1239
phylum
2

186801
class
2

1
2

SRS1041116\_contig\_number\_2218
186802
order

species
1950888

SRS049896\_contig\_number\_31964
1
